# Microbiological Surveillance and State of the Art Technological Strategies for the Prevention of Dialysis Water Pollution

**DOI:** 10.3390/ijerph9082758

**Published:** 2012-08-02

**Authors:** Piergiorgio Bolasco, Antonio Contu, Patrizia Meloni, Dorio Vacca, Andrea Galfrè

**Affiliations:** 1 Territorial Department of Nephrology and Dialysis, ASL, Quartu Sant’Elena, Cagliari 09045, Italy; Email: dialisi.territorio@asl8cagliari.it; 2 University Department of Public Health, Clinical and Molecular Medicine, University of Cagliari, Cagliari 09121, Italy; Email: acontu@unica.it (A.C.); lia@unica.it (P.M.); doriovacca@gmail.com (D.V.)

**Keywords:** microbiological methods, quality dialysis water

## Abstract

*Methods:* The present report attempts to illustrate the positive impact on the microbiological quality of dialysis patients over a 15-year period through the progressive implementation of state-of-the-art technological strategies and the optimization of microbiological surveillance procedures in five dialysis units in Sardinia. *Results:* Following on better microbiological, quality controls of dialysis water and improvement of procedures and equipment, a drastic improvement of microbiological water quality was observed in a total of 945 samples. The main aim was to introduce the use of microbiological culture methods as recommended by the most important guidelines. The microbiological results obtained have led to a progressive refining of controls and introduction of new materials and equipment, including two-stage osmosis and piping distribution rings featuring a greater capacity to prevent biofilm adhesion. The actions undertaken have resulted in unexpected quality improvements. *Conclusions:* Dialysis water should be viewed by the nephrologist as a medicinal product exerting a demonstrable positive impact on microinflammation in dialysis patients. A synergic effort between nephrologists and microbiologists undoubtedly constitutes the most effective means of preventing dialysis infections.

## 1. Introduction

In Europe, patients receiving hemodialysis undergo at least three treatment sessions a week. As standard hemodialysis treatment sessions last 4–6 hours, individual patients are exposed to 15,000–20,000 L of dialysis fluid yearly. In hemodiafiltration procedures, dialysis water is administered in the form of dialysate and infusate up to 3,400–6,800 L directly following the ultrafiltration through two or three dialysis water monitor ultrafilters [1]. The increasing tendency to use on-line hemodialysis procedures implies therefore the potential presence in distribution rings of microbial, fungal, and chemical substances which should be carefully monitored, as provided for in specific guidelines [2,3]. Moreover, waters should be tested by certified laboratories using significantly different analytical procedures, particularly in the case of microbiological controls, to those applied to test human blood and secretions [3]. Numerous undesirable substances originate from polluted water or infiltrations in the drinking water supply network. Humans are the major culprits, contaminating waterworks through the use of ineffective measures to reduce bacterial and/or chemical pollutant loads originating from surface waters collected in artificial basins, or from the building of drinking water networks using obsolete or inappropriate materials. The majority of toxic contaminants derive from procedures applied in the treatment of municipal waters, thereby suggesting that these are not always safe for direct use in hemodialysis applications. An unmonitored dialysis water treatment facility, an inefficient system for the distribution of dialysis waters to monitors, or a scarce regularity in disinfection constitute the most frequent causes of harmful or fatal substances which may prove to be particularly hazardous when adopting on-line procedures lacking effective ultrafilters with administration. Thus, the main parameters to be applied in the treatment of waters destined for use in hemodialysis procedures, should be underlined.

As a premise enhancing evaluation of the evolution of the plants implemented, a synthesis of the most recent ANSI/AAMI/ISO guidelines published in 2009 and 2011 is provided [2,3,4,5]. The Authors wish to underline that dialysis procedures used in the USA do not foresee the administration to patients of such a high number of infusions as occurs in Europe, when the nephrologist prescribes high convective methods such as hemodiafiltration. Furthermore, two-stage reverse osmosis (TRO) with overnight thermal disinfection is referred to, although not described in detail, in the AAMI Guidelines, and is to date applied scarcely throughout Italy and Europe.

Water utilized in hemodialysis applications is prepared in a series of different purification processes, the most common of which include [3,4,5,6,7,8]: reverse osmosis, deionization, and carbon filtration. Reverse osmosis (RO) uses high pressure to force water across a semi-permeable membrane to form product water (permeate), thereby rejecting 90 to 99 percent of ionic contaminants and >95 percent of non-ionic contaminants. A RO membrane is an effective barrier against microbiological contaminants, bacteria, viruses, and endotoxins. Currently however, two-stage reverse osmosis is actually the most effective form compared to one-stage reverse osmosis (SRO), achieving the highest degree of contaminant removal. This procedure is particularly recommended for use in on-line hemodialytic methods with high convective component [9]. Deionization (DI) uses ion exchange resins to remove ionic contaminants from water by exchanging hydrogen ions for cationic resins and hydroxyl ions for anionic resins. Mixed bed deionizers contain both cationic and anionic resins but do not remove microbiological contaminants. Deionizer performance should be closely monitored to avoid exhaustion, and it should be taken into account that ions bind to the resin with low affinity. This phenomenon, which may lead to high contaminant concentrations in the product water as the deionizer becomes exhausted [10], has been reported to cause acute, fatal fluoride toxicity in hemodialysis patients [11]. This method is virtually obsolete in Italy. In the pre-treatment procedures a variety of filters may be utilized, both as pre-treatment for main tap water purification processes and at the end of the pre-treatment system to remove coarse particulate matter prior to purification and to protect RO membranes from fine particles washed out of carbon beds. The use of chlorination and dechlorination is not mandatory, although in a few circumstances (e.g., during the drought in Sardinia in 1991) municipal authorities have implemented provisions including water supply on alternate days, and consequent reduced pressure throughout the water supply networks, which were frequently old and leaking (personal observation); this resulted in an infiltration of polluted waters from the water table in the vicinity of tubes conveying the drinking water supply. It should moreover be underlined that in Italy the use of underground water tables in supplying water to hospitals is permitted. Good quality proportional pumps should be applied to guarantee optimal chorine concentration (0.5–1 ppm), although complete water dechlorination should be ensured upstream of osmosis membranes. To prevent inadvertent exposure of patients to chloramine as the capacity of the carbon is exhausted two carbon filters in series are necessary. Inadequate chloramine removal may occur however, and assays aimed at detecting the presence of chloramines with specific rapid water checks are mandatory [12].

In water softeners resin beads inside the tanks display a high affinity for bivalent cations (calcium and magnesium) present in the feed water, releasing two sodium ions (monovalent) for each calcium or magnesium ion captured to prevent RO membranes from fouling by calcium and magnesium salts. Softeners must be regenerated by exposing resin to strong brine with a very high sodium concentration. Dechlorinators and softeners represent a suitable “pabulum” for bacterial growth due to the impossibility of disinfecting the same with a specific product unless thorough rinsing is undertaken against the flow. The treated water exits the central water treatment plant and is distributed to hemodialysis monitor by piping ring with an optimal diameter to avoid sluggish segments; metals such as brass, aluminium, or galvanized metal should not be used, and the distribution system must be designed as a closed ring. The use of polyvinyl chloride (PVC) should be eliminated and materials such as high corrosion resistance stainless steel (INOX AISI 316L) polyethylene thermoplastic polymer (PEX), and polyvinylidenefluoride (PVDF) used to construct piping and recently the polytetrafluoroethylene (PTFE) has been introduced.

The pollutant potential of reservoir storage is frequently underestimated. Tanks should be opaque, made of plastic for foodstuffs, and not be located in a “stagnant” corner of the circuit, but should guarantee a constant flow of water. However, a fundamental aspect is constituted by the bacteriological and endotoxin controls to be implemented in line with appropriate procedures in sampling and transportation, in inoculation of the culture medium with dialysis water, and in the accurate interpretation of results, not taking into account merely mesophiles from the environment, but furthering observation to evaluate the presence of bacteria or mycetes [13]. Based on these premises therefore, a study was undertaken to assess the quality of water in five dialysis units in which over 16 years numerous changes had been implemented in methods used for disinfection and monitoring and improvements made to the equipment used in the production and distribution of ultrapure dialysis water, particularly in dialysis units adopting online convective procedures with production of high volumes of infusate, such as online hemodiafiltration techniques.

## 2. Materials and Methods

Characteristics of the units: five dialysis units were investigated over a 16 year 3 month period (195 months) from May 1996 to April 2012. In total 945 samples were taken (minimum three samples for each distribution ring every three months in steady state equipment). The observation periods are illustrated in Table 1, Table 2 and Table 3.

### 2.1. Use of Polyvinyl Chloride (PVC) Piping: Two Centres—See Table 1

PVC piping was used in two successive periods:

1. Use of irregular/occasional microbiological controls, chemical disinfection and SRO.
2. Use of regular microbiological controls, chemical disinfection and SRO in polyamide. The use of PVC however was discontinued and it was replaced with PEX rings in one unit and AISI INOX 316L steel rings in the other.

Student’s T-test was used for comparing data. Results were considered significant when *p* was <0.05.

**Table 1:** PVC piping.

Two dialysis centres	Occasional chemical disinfection (average every quarter)	Regular monthly disinfection
Reverse osmosis	SRO	TRO
Technical dialysis posts	29	29 ^a^
Observation time, months	60	36
Sampling, total numbers	120	72
CFU, mL^–1^, average, bacteria *	238.7 ± 374.3	153.9 ± 160.7
CFU, mL^–1^, average, mycetes *	19.2 ± 34.4 ^b^	5.2 ± 7.5 ^c^
CFU, mL, medianbacteria	122.2	25.5
CFU, mL, minimum and maximum values bacteria	7–1,773	0–1,500

^a^ Six posts switch to INOX and another 23 switch to PEX piping (Table 2 and Table 3); the PVC piping ring was abandoned due to its inadequate safety; ^b^ *vs.* ^c^: *p* < 0.05; (*) values represent averages ±1 standard deviation from (heterotrophic plate count) HPC 22 °C measurements. Average and median are both derived from bacteria and mycetes at HPC 22 °C.

### 2.2. Use of AISI INOX 316L Piping: Three Centres—See Table 2

We used high corrosion resistance stainless steel piping in three successive periods:

1. Use of AISI INOX 316L steel distribution ring with irregular/occasional microbiological controls, chemical disinfection and one stage reverse osmosis (SRO) system in polyamide;
2. Use of AISI INOX 316L steel distribution ring with regular microbiological controls, chemical disinfection and SRO system in polyamide;
3. Use of AISI INOX 316L steel distribution ring with regular microbiological controls and daily overnight pulsed thermal disinfection by means of TRO.

**Table 2:** AISI INOX 316L piping.

Three dialysis units	Occasional chemical disinfection (average every quarter)	Regular monthly chemical disinfection	Regular daily thermal disinfection
Reverse osmosis	SRO	SRO	TRO
Technical dialysis posts	21	27 ^a,b^	27
Observation time, months	60	87	36
Sampling, total numbers	180	261	108
CFU, mL/min, average, bacteria	160.4 ± 321.1 ^1^	43.1 ± 76.1 ^2^	15.0 ± 24.6 ^3^
CFU, mL/min, average, mycetes	35.8 ±53.9 ^4^	8.1 ± 8.7 ^5^	1.75 ± 0.41 ^6^
CFU, mL, median bacteria	19.3	21.2	0
CFU, mL, minimum and maximum values bacteria	0–520	0–107	0–2

^a^ Six piping rings of PVC (Table 1); ^b^ increase of technical dialysis posts due to enlargement of two dialysis centers; Significance: ^1^ *vs*. ^2^ and ^3^ *vs*. ^1^, ^2^: *p* < 0.03; ^4^ *vs*. ^5^, ^6^: *p* < 0.02.

### 2.3. Use of PEX (Polyethylene Thermoplastic Polymer) Piping: Two Centres—See Table 3

PEX piping was used in three successive periods:

1. Use of PEX distribution ring with irregular microbiological controls, occasional chemical disinfection and SRO system in polyamide;
2. Use of PEX distribution ring with regular microbiological controls, chemical disinfection and SRO system in polyamide;
3. Use of PEX distribution ring with regular microbiological controls and daily overnight pulsed thermal disinfection by means of TRO.

**Table 3:** PEX piping.

Two dialysis units	Occasional chemical disinfection (average every quarter)	Regular monthly chemical disinfection	Regular daily thermal disinfection
Reverse osmosis	mono-osmosis	mono-osmosis	bi-osmosis
Technical dialysis posts	23 ^d^	23 ^d^	31 ^e^
Observation time, months	24	24	36
Sampling, total numbers	48	48	72
FU, mL/min, average, bacteria	398.1 ± 627 ^1^	33.7 ± 29.9 ^2^	2.4 ± 2.3 ^3^
CFU, mL/min, average, mycetes	18.4 ± 38.8 ^4^	0.37 ± 0.84 ^5^	0.30 ± 0.4 ^6^
CFU, mL, median bacteria	101	19.1	0.5
CFU, mL, minimum and maximum values bacteria	0–1,028	0–75	0–0.9

^d^ 23 piping rings of PVC (Table 1); ^e^ increase of technical dialysis posts due to enlargement of two dialysis centers; Significance ^1^ *vs.* ^2^, ^3^ and ^3^ *vs.* ^1^, *p* < 0.01; Significance ^4^ *vs*. ^5^, 6: *p* < 0.01.

### 2.4. Brief Description of Characteristics of Materials and Operations

#### Pre-Treatment

Hard water should undergo mandatory treatment with double water softeners. Softeners are sized in grains of capacity and are invariably regenerated on a routine daily basis with concentrated sodium chloride solution (brine). This method was applied throughout the observation study.

Dechlorination represents another fundamental step. We used tanks containing approximately 400 L granular activated carbon (CAG) which had never been regenerated. Throughout the first five years of operation the carbon beds had not been replaced, although in the last seven years CAG had been changed on a yearly basis and carbon tanks subjected to daily back-washing. Softeners and dechlorinators cannot be disinfected. The product commonly applied in disinfection may damage their material beds. The amount of softeners used in the units investigated was invariably overestimated, in order to guarantee an effective softening of inflow water featuring >1,000 mS/cm.

One-stage reverse osmosis. Thin film RO membranes are made from polyamide and spiral wound around a permeate-collecting tube. This material is compatible with peracetic acid used in disinfection. Mono-osmosis techniques have been abandoned over the last three years and substituted by TRO. The new CWP WRO 131/132 ROHH Gambro^®^ equipment with ISO 14021 (using polyamide membranes) adopted in the latter stage, comprising a completely automated system of thermal disinfection that no longer required the use of chemical disinfection products, featured an integrated system for the thermal disinfection of membranes and distribution rings. Thermal disinfection of biosmosis systems provides for an automated daily thermal disinfection of dialysis room distribution circuit using water heated to 90/95 °C, to prevent the formation of biofilms. The heated disinfection unit, integrated in the biosmosis plant, is programmed to heat water from 63 °C to 90/95 °C at a pre-specified time of day, towards the end of the dialysis session with the possibility of varying programming for each day of the week. The circulation of hot water in the dialysis room distribution circuit occurs at the end of the dialysis session and continues throughout the night until shortly before the start of the morning session when piping temperatures return to non-dangerous levels. In addition to providing correct operating parameters, the integrated computer in the TRO system records data for the last three operating sessions and elaborates these data as required, in the form of text, synoptic diagram or a graph. 

Chemical disinfection of distribution ring downstream of dialysis monitors was carried out on a monthly basis and was abandoned following the introduction of thermal disinfection.

The connection between distribution ring and monitors remains a highly critical and somewhat underestimated aspect. Use of a mesh tube has been definitely prohibited. A mesh tube constitutes an ideal *pabulum* for the growth of bacteria, mycetes, and algae. Approximately 15 days later swabs were obtained from the inside of mesh tubes, yielding significant results for microbiological load >10,000 CFU/cm^2^ (personal observation), and were subsequently replaced at all kidney positions by large extendable PEX springs suitable for monthly disinfection using peracetic acid and/or chlorine oxidant. 

### 2.5. Microbiological and Endotoxin Controls

#### Assumption

In agreement with microbiologists, the authors opted to extend tests to a higher spectrum of micro-organisms frequently detected in previous studies, particularly as the hydro-geographic situation in Sardinia is almost exclusively characterised by the presence of surface waters collected in artificial basins. Indeed, in view of the latter and to the high temperatures registered during summer months, the Italian Department of Health has not ruled out the potential development of harmful bacteria, including cyanobacteria [14] resulting in the release of cyanotoxins. Preparations are therefore currently being made to assemble procedures for use in detecting the latter. 

Sampling is carried out according to a series of procedures aimed at allowing an appropiate aliquot of water to be obtained for analysis. Depending on the test to be performed, prior to sampling the volume of water required for testing should be ascertained. Bottles and vials used to collect samples for microbiological analysis should never be washed at time of sampling. In addition to exposing recipients to possible contamination, rinsing would remove traces of sodium thiosulphate present in the tap. Water should be left to run long enough to eliminate disinfectants prior to sampling. 

Taps should be cleaned and disinfected prior to sampling; both taps and the inside of collars should be cleaned and residues, dust, mucilage, detergents and disinfectants, and other substances capable of affecting the outcome of microbiological analysis should be removed. A solution of sodium hypochlorite or similar types of disinfectant should be used in cleansing: 10% solutions of commercially available sodium hypochlorite or sodium dichloroisocyanurate may be used. Due to the corrosive nature of these products, particular caution should be applied in using the solutions. Fatty deposits should be removed by rubbing with isopropyl alcohol. The disinfectant solution should be left on the taps for 2–3 minutes, and all traces of the product subsequently removed. It is fundamental that during sampling procedures contamination should be avoided and the quality of water sample maintained. In addition to the mandatory cleansing and disinfection procedures, metal taps may also be flame gouged. However, if the latter procedure is performed in a perfunctory manner, it will not produce the desired effect on potential microbial contamination. As previously, the tap should be left to run for 2–3 minutes and flow left untouched during sample collection. On collection, open the sterile bottle taking care to not touch the inside of the cap or the inside of the bottle neck which will come into direct contact with the sample, and close immediately after sampling. Particular care should be taken to not overfill the bottle in order to enhance effective lab homogenisation of the sample at the time of testing.

### 2.6. Transportation

During transportation and storage of samples for microbiological testing, the representative nature of samples should be ensured by preventing regrowth of micro-organisms. As far as possible alterations which may at times be inevitable in a small aliquot of water stored in a closed container, should be kept to a minimum. The sample should be stored away from the light (both ultraviolet and visible) and from high temperatures, and appropriate conditions of hygiene applied during transportation. From the time of sampling to arrival in the lab, all samples should be stored at a temperature of below 10 °C, with the optimum range of 2–8 °C being recommended. The samples must be inseminated within two hours (ISO 19458:2006). Between sampling and the analysis, the maximum time for the microbiological analysis depends on the type of analysis or better by the parameters to be searched as shown in the Table 4.

**Table 4:** Maximum time of analysis between sampling and analysis.

Parameter	Maximum time of analysis in hours
Total bacterial count at 22 °C or 37 °C	8–12
*Escherichia coli* and coliforms	12–18
*Enterococci*	12–18
Bacteria and spores of sulphite-reducing clostridia	48–72
*Pseudomonas aeruginosa*	8–12

### 2.7. Analytical Procedures

In the Authors’ opinion, microbiological testing procedures should not only assess the presence of environmental mesophiles and mycetes, but should also address the issue of detecting specific pathogens. For each of microbiological procedures we referred to ISO 13959:2009 and (UNI EN ISO 6222:2001) [2,13] for the different bacteria in order to obtain further implemented improvements and applying the specific Italian methodologies described in the sections below [15,16,17]. We also have considered the procedures applied by other authors [18,19]. The choice of parameters to test depends in particular on the water supply system. In our case over 90% of drinking waters are surface water, resulting in poor water quality. In addition, losses of about 35–40% are observed in most of the water network. Given the precariousness of the sample, also associated with the low concentration of free chlorine residual detected (0:01 to 0:02 mg/L), and the use for which it is intended, it was considered important to search also indicators of faecal contamination in countries where surface water is used for drinking water production.

#### 2.7.1. Total Coliforms

An aliquot (100 mL) of the sample collected should be filtered through a 47 mm cellulose ester membrane with filtration characteristics corresponding to a nominal pore size of 0.45 µm. Place the membrane over the M-Endo agar LES medium and incubate at 36 ± 1 °C for 18–24 hours. Red-coloured colonies with a metallic sheen that grow over a 24-hour period, generally accompanied by a dark brick-red colouring in the area beneath the membrane, are deemed to be coliform bacteria (presumed coliforms). Tests should be carried out on colonies aged no more than 24 hours.

Tests should then be undertaken to verify coliform status: the presence of the cytochromoxydase enzyme on all, or at least a representative number of typical colonies should be assessed and biochemical identification tests performed (UNI EN ISO 9308-1:2002) [20]. Tests should be carried out on colonies aged no more than 24 hours.

#### 2.7.2. *Enterococci*

An aliquot (100 mL) of the sample collected should be filtered through a 47 mm cellulose ester membrane with filtration characteristics corresponding to a nominal pore size of 0.45 µm. Place the membrane over the Slanetz and Bartley agar medium and incubate at 36 ± 1 °C for 40–48 hours. Following incubation, colonies ranging from pink to dark red and brown (in the centre or throughout the colony) are deemed typical (presumed *Enterococci*). Tests should be undertaken to confirm enterococcus status: the presence of the catalase enzyme and the esculin hydrolysis test should be undertaken on all, or at least a representative number of typical colonies and biochemical identification tests performed. Tests should be carried out on colonies aged no more than 24 hours (UNI EN ISO 7899-2: 2003) [20].

#### 2.7.3. *Pseudomonas aeruginosa*

An aliquot (250 mL) of the sample collected should be filtered through a 47 mm cellulose ester membrane with filtration characteristics corresponding to a nominal pore size of 0.45 µm. Place the membrane over the Pseudomonas agar/CN medium and incubate at 36 ± 1 °C for 40–48 hours After (2 ± 2) and (44 ± 4) hours assess the growth of typical colonies on isolation medium. *Pseudomonas aeruginosa* may develop as greenish-blue colonies that produce pyocyanin, fluorescent or reddish-brown colonies. Count all characteristic colonies (presumed to be *Pseudomonsas aeruginosa*). Tests should then be undertaken to confirm *Pseudomonsas aeruginosa* status: place under a Woods lamp and following observation on all, or at least a representative number of typical colonies, biochemical identification tests should be performed. Tests should be carried out on colonies aged no more than 24 hours (UNI EN ISO 12780:2002) [20].

#### 2.7.4. *Clostridium perfringens*

An aliquot (100 mL) of the sample collected should be filtered through a 47 mm cellulose ester membrane with filtration characteristics corresponding to a nominal pore size of 0.45 µm. Place the membrane over the Tryptose Sulfite Cycloserine Agar medium, and cover the membrane completely with 5–6 mL of liquid culture medium at a temperature of 50 ± 5 °C. Leave to set and incubate at a temperature of 44 ± 1 °C for 21 ± 3 hours under anaerobic conditions. Micro-organisms belonging to the *Clostridium perfringens* species form black or grey/yellowish-brown colonies on the Tryptose Sulfite Cycloserine Agar medium, conveying a slightly darker shade above or below the filtrating membrane. Count all typical colonies and treat as presumed *Clostridium perfringens*. Isolate colonies on two parallel blood Agar plates and incubate one plate under anaerobic conditions and the other under aerobic conditions at 36 ± 1 °C for 21 ± 3 hours. Following incubation, examine plates to check the presence or absence of growth. *Clostridium perfringens* colonies typically produce areas of clear hemolysis on culture medium. Undertake confirmation tests only on colonies growing on blood Agar under anaerobic conditions, and subsequently perform biochemical identification tests. Tests should be carried out on colonies aged no more than 24 hours. Method ISS A 005B REV.00 (National Standard Method W 5 ISSU 3 dell’Health Protection Agency).

#### 2.7.5. Mycetes

An aliquot (100 mL) of the sample collected should be filtered through a 47 mm cellulose ester membrane with filtration characteristics corresponding to a nominal pore size of 0.45 µm. Place the membrane over a Sabouraud Dextrose Agar medium and incubate at 22–25 °C for 3 ± 5 days. Recognition of fungal species is a complex issue requiring lengthy observation and proven experience. The aspect of colonies varies according to the type of substrate (natural or artificial) and colonies develop on the basis of numerous factors. To identify yeasts sample from the centre of the colony and smear on to a glass slide in a drop of sterile distilled water. View under a microscope, preferably under contrast and at 20× or 40× magnification to identify pseudomycelium and spores; if necessary, perform commercially available miniaturised biochemical identification tests (UNI EN ISO 6222:2001). Use direct observation technique to view filamentous fungi under the microscope. Carefully attach a piece of transparent sticky tape to the surface of the colony to be examined; transfer the tape with the sticky part facing downwards, onto a glass slide containing a drop of lactophenol blue solution and cover with a glass cover slip; observe at 100X to enhance distinguishing of fructiferous bodies [20,21].

#### 2.7.6. Counting of Colonies 22 °C–37 °C

Analytical procedures undertaken to assess the number of micro-organisms at 37 °C and 22 °C are identical for both parameters, comprising the agar inclusion technique. Seed 1 mL of the sample onto the bottom of a Petri dish. Pour approximately 15 mL of isolation substrate maintained in a liquid state at a temperature of 45 ± 1 °C on to the dishes containing the inoculum. Mix thoroughly by rotating dishes backwards and forwards to facilitate a complete mixing of culture medium and sample. Leave to set and incubate one dish at 36 ± 1 °C for 40–48 hours, and the other at 22 ± 1 °C for 64–72 hours (UNI EN ISO 6222:2001) [20].

#### 2.7.7. Counting at 22 °C (Mesophiles) (UNI EN ISO 6222:2001)

An aliquot (100 mL) of the sample collected should be filtered through a 47 mm cellulose ester membrane with filtration characteristics corresponding to a nominal pore size of 0.22–0.45 µm. Place the membrane on the agar medium and incubate at 20–23 °C for 7 days (UNI EN ISO 6222:2001) ) [20]. 

### 2.8. Endotoxin Tests

Tests are carried out both on osmotic water as monitors are detached [22] and at random initial-intermediate and end points in the ring. Spectrophotometric methods are used (Endosafe^®^-PTS™, Charles River^®^) with gel-clotting at 37 °C. Acceptable limits of detection on ring detachment are <0.05, and <0.01 for online infusion liquid. A training course in use of this piece of equipment is required, the machine should be calibrated by the manufacturers on a yearly basis. Indispensable requisites for on-line hemodiafiltration are as follows: CFU: 0 colonies/mL bacteria and mycetes, endotoxins <0.03 EU/mL. This method should not be used if the requisites are lacking.

## 3. Results

Samples were obtained as far away as possible from the most recent point of disinfection according to a rotation schedule between at least three locations situated at the beginning, the centre and the end of the ring; cultured dishes were read as provided for in standard guidelines. Significance difference was observed adopting a microbiological improvement strategies and plants as illustrated in Table 1, Table 2 and Table 3 showing the experience gained throughout the period of observation.

## 4. Discussion

The Authors’ experience in five territorial dialysis units, implemented on the basis of microbiological results obtained at several points throughout the central plant and piping ring, have led to an improvement in microbiological/endotoxinic quality of water subsequent to changes made in the use of materials and procedures. Specifically, it has been demonstrated how, in the presence of materials other than PVC, regular disinfection and frequent controls (even monthly) represented a fundamental step forward in achieving low levels of CFU [23]. The use of PVC should be avoided; it is not suited for use with thermal disinfection methods and evidence has indicated it’s potentially cancerogenic status [24]. The overnight thermal disinfection achieved by means of biosmosis resulted in a dramatic decrease in microbial load [7], which appeared to be more significant following the use of PEX compared to INOX in construction of the distribution ring. In the Authors’ opinion, this difference may be associated to diameter of piping; a minor diameter decreased the possibility of stagnant areas creating a less suitable habitat for biofilm growth. In fact it is fundamental that pressure along the walls, and velocity and intensity of fluid shear stress on the smaller inner surfaces, are higher than those recorded in larger-sized sections; in other words a higher water pressure and velocity enhance filling of the circuit and ensure against the formation of concealed areas where bacteria and hyphae may flourish. Moreover, frequent thermal disinfection does not rapidly prevent the formation of bacterial biofilm and hyphae along inner surfaces. Therefore, in the wake of experience gained in units operating with PVC rings, the Authors opted to abandon use of this material entirely. The application of meticulously implemented periodic thermal and chemical disinfection procedures should be further supported by additional checks and immediate correction of any “pathological” areas detected by microbiological results. Accordingly, a specialized team should be appointed to carry out controls and periodic and non-scheduled maintenance based on the finding of microbiological levels exceeding established safety limits.

Irrespective of the material used in construction of the distribution ring, the connection valves to dialysis monitors should be of stainless steel AISI 316L, in view of the potential liability of the connection and the increased risk of stagnant areas at this level: this option results in a decreased probability of microbial adhesions. In all the processes described, chemical, physical and bacteriological tests should be undertaken by a certified laboratory. The Authors maintain that standard procedures should be established in conjunction with an institution specializing in environmental water testing; labs should moreover possess a working knowledge of the equipment used and ensure that methods described in the leading guidelines in the field adhered to. A synergic action between the dialysis unit staff, the maintenance team and the lab is mandatory, with purpose-trained specialised technicians performing sampling throughout all units and providing for a correct transportation to the testing lab.

The Authors underlined how systems should be monitored continuously and the following taken into account [25]: (a) on opting for the sole chemical disinfection, apply every 15–30 days solutions capable of removing both bacterial biofilms and potential mineral scales that may protect bacteria and/or enhance their survival; (b) daily nocturnal thermal disinfection; to promote safety, an additional monthly or twice monthly disinfection of osmosis membranes and ring as described in item. 

The experience gained has led the Authors to progressively undertake alterations aimed at optimizing the microbiological quality of dialysis water: weak points may be represented by an excessive inner diameter of piping, both in PVC and INOX, resulting in pipes that are disproportionate to water flow and volume; the definitive abolishing of PVC and mesh tubes connecting the ring to monitors is fundamental.

Conversely, the strong points that emerged from the present study focused largely on the use of PEX. However, very few literature reports published to date have focused on the efficacy of the latter material in preventing the adhesion of bacterial biofilms. Stainless steel INOX AISI 316 L is a long-lasting but rather costly material; the length of duration however may promote the amortization of costs. On opting to use stainless steel assembly and soldering should be undertaken by specialized technicians in order to warrant a perfect connection between the various segments. The studies undertaken with stainless steel were perfectly satisfactory; however, the Authors prefer to underline the suitability of PEX circuits which have no seams and, according to the quality of water, provide for a scheduled replacement of the circuit every 7–10 years. The importance of applying a correctly-sized piping and an antireflux gooseneck drainage spout should be underlined. 

## 5. Conclusions

The carrying out of a regular and scrupulous microbiological surveillance enables physicians and microbiologists to identify the faulty part of equipment and/or ring piping and implement the required improvements to materials, equipment, and disinfection procedures. Indeed, this result was achieved in the territorial dialysis centres investigated in the present study, all of which featured significantly diverse hydrological and geographic differences. 

Over a 16-year period the dynamic procedures set up have demonstrated how TRO should be considered a necessary investment to be faced with the aim of establishing a safer microbiological profile and producing a positive impact on microinflammation in dialysis patients, particularly in the case of increased infusion volumes during on-line hemodiafiltration procedures [26]. Likewise, daily overnight thermal disinfection procedures have proved at times to be more effective than frequent chemical disinfection. 

Finally, it has become increasingly clear how staff operating in nephrology and microbiology units should possess a strong, motivated cultural synergic background on this complex subject in order to supply the best possible quality water to consumers. Indeed, dialysis water today should be considered a “medicinal product” in its own right.

## References

[1] Locatelli F., Altieri P., Andrulli S., Bolasco P., Sau G., Pedrini L.A., Basile C., David S., Feriani M., Montagna G. (2010). Hemofiltration and hemodiafiltration reduce intradialytic hypotension in ESRD. J. Am. Soc. Nephrol..

[2] International Organization for Standardization (IOS) (2009). Water for Haemodialysis and Related Therapies; ISO 13959.

[3] International Organization for Standardization (IOS) (2009). Dialysis Fluid Quality for Haemodialysis and Related Therapies; ISO 11663.

[4] International Organization for Standardization (IOS) (2009). Water Treatment Equipment for Haemodialysis and Related Therapies; ISO 26722.

[5] Martin K., Laydet E., Canaud B. (2002). European best practice guidelines for haemodialysis (Part 1). Nephrol. Dial. Transplant..

[6] Alloatti S., Bolasco P., Canavese C., Cappelli G., Pedrini L., Pizzarelli F., Pontoriero G., Fuiano G. (2005). Guidelines on water and solutions for dialysis. Italian Society of Nephrology. G Ital Nefrol..

[7] Bolasco P., Scotto P. Dialysis Fluid Purity in Hemodialysis: Normal Values and Undesirable Substances. Proceedings of CIN 2011 6th Congress of Nephrology in Internet.

[8] International Organization for Standardization (ISO) (2011). Guidance for the Preparation and Quality Management of Fluids for Haemodialysis and Related Therapies; ISO 23500.

[9] Pontoriero G., Pozzoni P., Andrulli S., Locatelli F. (2003). The quality of dialysis water. Nephrol. Dial. Transplant..

[10] Bland L.A., Arnow P.M., Arduino M.J., Bova J., McAllister S.K. (1996). Potential hazards of deionization systems used for water purification in hemodialysis. Artif. Organs..

[11] Arnow P.M., Bland L.A., Garcia-Houchins S., Fridkin S., Fellner S.K. (1994). An outbreak of fatal fluoride intoxication in a long-term hemodialysis unit. Ann. Intern. Med..

[12] Smith M.P., Marr F.E., Kanagasundaram N.S. (2011). Chloramine reduction by reverse osmosis membranes. Hemodial. Int..

[13] Ray J. (2007). Microbiological monitoring of dialysiswater systems—Which culture method?. J. Ren. Care..

[14] Azevedo S.M., Carmichael W.W., Jochimsen E.M., Rinehart K.L., Lau S., Shaw G.R., Eaglesham G.K. (2002). Human intoxication by microcystins during renal dialysis treatment in Caruaru-Brazil. Toxicology.

[15] (2007). Reference Analytical Methods for Drinkable Water Second Law by Decree 31/2001; Chemical Reports ISTISAN07/5.

[16] (2007). Reference Analytical Methods for Drinkable Water Second Law by Decree 31/2001; Microbiological Reports ISTISAN07/5.

[17] (2003). Guidelines for Environmental Infection Control in Health-Care Facilities; Recommendations of Practices Advisory Committee (HICPAC) CDC and the Healthcare Infection Control.

[18] Arvanitidou M., Vayona A., Spanakis N., Tsakris A. (2003). Occurrence and antimicrobial resistance of Gram-negative bacteria isolated in haemodialysis water and dialysate of renal units: Results of a Greek multicentre study. J. Appl. Microbiol..

[19] Laurence R.A., Lapierre S.T. (1995). Quality of hemodialysis water: A 7-year multicenter study. Am. J. Kidney Dis..

[20] Köster W., Egli T., Ashbolt N., Botzenhart K., Burlion N., Endo T., Grimont P., Guillot E., Mabilat C., Newport L., Niemi M., Payment P., Prescott A., Renaud P., Rust A. (2003). Analytical Methods for Microbiological Water Quality Testing. Assessing Microbial Safety of Drinking Water: Improving Approaches and Methods.

[21] Pires-Gonçalves R.H., Sartori F.G., Montanari L.B., Zaia J.E., Melhem M.S., Mendes-Giannini M.J., Martins C.H. (2008). Occurrence of fungi in water used at a haemodialysis centre. Lett. Appl. Microbiol..

[22] Pizzarelli F., Cerrai T., Biagini M., Malaguti M., Bargagna R. (2004). Dialysis water treatment systems and monitoring in Italy: Results of a national survey. J. Nephrol..

[23] Bambauer R., Schauer M., Jung W.K., Daum V., Vienken J. (1994). Contamination of dialysis water and dialysate. A survey of 30 centers. ASAIO J..

[24] Pirastu R., Comba P., Reggiani A., Foa V., Masina A., Maltoni C. (1990). Mortality from liver disease among Italian vinyl chloride monomer/polyvinyl chloride manufacturers. Am. J. Ind. Med..

[25] Cappelli G., Ravera F., Ricardi M., Ballestri M., Perrone S., Albertazzi A. (2005). Water treatment for hemodialysis: A 2005 update. Contrib. Nephrol..

[26] Canaud B. (2011). The early years of on-line HDF: How did it all start? How did we get here?. Contrib. Nephrol..

